# Evaluation of Mental Health First Aid from the Perspective Of Workplace End UseRs—EMPOWER: protocol of cluster randomised trial phase

**DOI:** 10.1186/s13063-020-04636-0

**Published:** 2020-08-14

**Authors:** Opeyemi Atanda, Patrick Callaghan, Tim Carter, Graham Durcan, Nick O’Shea, Steve D. Brown, Paula Reavey, Eleni Vangeli, Sarah White, Kerry V. Wood

**Affiliations:** 1 London South Bank University, London, UK; 2 University of Nottingham, Institute of Mental Health and School of Health Sciences, Nottingham, UK; 3 Centre for Mental Health, London, UK; 4 Nottingham Business School, Nottingham Trent University, Nottingham, UK; 5 Population Health Research Institute, St George’s University of London, London, UK

**Keywords:** Mental health first aid, Help seeking behaviours, Workplace, Process evaluation, Economic evaluation

## Abstract

**Background:**

Mental Health First Aid (MHFA) is a mental health intervention that teaches people how to identify, understand and help someone who may be experiencing a mental health issue. Reviews of the implementation of MHFA found between 68 and 88% of trained Mental Health First Aiders had used their skills when in contact with someone experiencing mental health difficulties. Reviews evaluating the impact of MHFA suggest positive outcomes. However, to date, there has been no systematic, rigorous evaluation of the impact of MHFA on recipients of the intervention, the organisations providing it and the cost-effectiveness of MHFA overall. This trial will evaluate the effectiveness and cost-effectiveness of MHFA.

**Methods:**

The study is a multi-centred, two-arm clustered randomised controlled trial. Organisations will be randomly allocated to the control or intervention (estimated sample size 800 recipients). The intervention is the standard MHFA intervention provided by Mental Health First Aid England (MHFAE). The control condition will be organisations having a brief consultation from MHFAE on promoting mental health and well-being in the workplace. The primary outcome is health seeking behaviour, measured using the Actual Help Seeking Questionnaire, at 6 months’ follow-up. Data collection will be undertaken at baseline (T0), post-intervention—up to 3 months (T1), at 6 months (T2), 12 months (T3) and 24 months (T4). The primary analysis will be conducted on those participants who receive MHFA, a per protocol analysis.

**Discussion:**

The study is the first to evaluate the effect of MHFA in the workplace on employees with direct and indirect experience of the intervention, when compared with usual practice. Being also the first to assess, systematically, the social impact of MHFA and investigate its cost-effectiveness adds to the originality of the study. The study promises to yield important data, as yet unknown, regarding the effectiveness, cost-effectiveness, implementation issues, and the sustainability of MHFA in the workplace.

**Trial registration:**

Clinicaltrials.govNCT04311203. Registered on 17 March 2020.

## Administrative information

**Table:** 

Title {1}	Evaluation of Mental Health First Aid from the Perspective of Workplace End Users – EMPOWER : Protocol of trial phase.
Trial registration {2a and 2b}.	Clinicaltrials.gov. NCT04311203
Protocol version {3}	Version 18.
Funding {4}	Mental Health First Aid England, 21 Prescot St, Whitechapel, London, E1 8BB. England.
Author details {5a}	Patrick Callaghan, Professor of Mental Health Science, Division of Psychology, School of Applied Sciences, London South Bank University, 02078157603, callaghanp@lsbu.ac.uk Paula Reavey, Professor of Psychology & Mental Health, Division of Psychology, School of Applied Sciences, London South Bank University, 02078156177, reaveyp@lsbu.ac.uk Steven D. Brown, Professor of Health and Organisations, Nottingham Business School, Nottingham Trent University, steven.brown@ntu.ac.uk Kerry V. Wood, Senior Research Fellow, Division of Psychology, School of Applied Sciences, London South Bank University, 02078155467, woodk6@lsbu.ac.uk. Dr Graham Durcan, Associate Director, Centre for Mental Health, Technopark, 90 London Road, SE1 6LN, 07957595593, Graham.Durcan@centreformentalhealth.org.uk Nick O’Shea – Chief Economist. Centre for Mental Health, Technopark, 90 London Road SE1 6LN, 07759 155177, nick.oshea@centreformentalhealth.org.uk Tim Carter, Assistant Professor in Mental Health, Room D25, Institute of Mental Health, School of Health Sciences , University of Nottingham, Triumph Road, Nottingham NG7 2TU, 0115 74 84318, timothy.carter@nottingham.ac.uk Eleni Vangeli, Senior Lecturer, Division of Psychology, School of Applied Sciences, London South Bank University, 02078155806, vangelie@lsbu.ac.uk. Opeyemi Atanda, PhD candidate, Division of Psychology, School of Applied Sciences, London South Bank University, atandao2@lsbu.ac.uk. Dr Sarah White, Statistician, Population Health Research Institute, St George’s University of London, London, UK. swhite@sgul.ac.uk
Name and contact information for the trial sponsor {5b}	London South Bank University, 103 Borough Road, London, SE1 0AA.
Role of sponsor {5c}	The design, conduct, data collection, analyses and interpretation of the trial are conducted by the researchers. Based on the findings, papers for publication will be prepared by the research team who will have ultimate authority over these activities. MHFAE CIC will assist with access to organisations prior to their recruitment into the study.

## Introduction

### Background and rationale {6a}

Mental Health First Aid (MHFA) is a mental health intervention on how to identify, understand and help someone who may be experiencing a mental health issue. It is designed to help Mental Health First-Aiders to listen, reassure and respond, even in a crisis—and potentially stop a crisis from happening. MHFA courses were developed in 2000 to equip trainees with first-aid skills to support people with mental health problems [1]. MHFA training is reported to increase trainees’ knowledge, confidence and skills in identifying mental health problems, as well as reducing negative attitudes and stigma [1–4].

Reviews of the implementation of MHFA found between 68 and 88% of trained mental health first aiders had used their skills when in contact with someone experiencing mental health difficulties [5–7]. Mental health interventions in the workplace have been linked to increased productivity, reduced levels of absenteeism and significant economic gains [8].

Recently, a large study (MENTOR) [9] investigated the implementation, use and utility of MHFA in the workplace using multiple research methods and reported that MHFA was useful for raising awareness of mental health in the workplace, but stated it may not be the best or only means of doing so and may not necessarily be the most cost-effective. The MENTOR study highlighted implementation issues relating to lack of clarity around the role of the mental health first aider and potential safety concerns, leading them to recommend further research and evaluation of the intervention. Implementation challenges such as role conflict relating to a person carrying out the first aider role versus other roles and responsibilities they may have within the organisation have been identified previously [10]. Handling the potentially hierarchical relationship between the mental health first aider and recipient, who may be a subordinate, is one such example of this potential role conflict [10].

Since the original research on MHFA, in 2001, there have been numerous studies evaluating MHFA among various populations, with benefits evident in adults and young people, across diverse organisations. However, there is limited evidence from studies conducted outside Australia, where the research was first conducted. Little evidence has shed light on the challenge of implementing MHFA in a range of organisations, and there is no evidence establishing the cost-effectiveness of MHFA, especially when compared to other ways of providing mental health and well-being support in the workplace. Few studies test the effect of MHFA directly on recipients. In addition, no empirical studies have directly investigated its wider social impact. MHFA appears to reduce mental health stigma in the workplace [1, 2, 9], and the potential of MHFA to reduce stigma and lower sickness absence and presenteeism in the workplace [8] speaks to wider social benefits such as keeping people in work and reducing discrimination. However, the lack of a social impact assessment of MHFA remains a significant gap in the evidence base.

In sum, evidence suggests MHFA benefits’ organisations by giving employees the tools to keep themselves and their colleagues healthy and facilitating a long-term cultural change across organisations. However, there has been no systematic investigation of the impact of MHFA on recipients of the intervention, the organisations that provide MHFA and the cost-effectiveness of MHFA. Furthermore, the wider societal impact of MHFA is not evident from the empirical literature.

### Objectives {7}

The EMPOWER study will implement a cluster randomised controlled trial (RCT) to examine the effectiveness and cost-effectiveness of MHFA in the workplace on help-seeking behaviours of employees, with embedded qualitative, process and social impact evaluations. In order to best answer questions regarding effectiveness, mechanisms and acceptability/feasibility, a design combining a RCT with a qualitative component will be adopted. The qualitative component will assess the efficiency, utility, usability, feasibility, acceptability and the mechanisms underlying the intervention. This protocol describes the trial element of EMPOWER.

### Trial design {8}

The study is a multi-centred two-arm cluster RCT comparing organisations implementing MHFA (intervention arm) with a consultation on mental health and wellbeing in the workplace (control arm).

## Methods: participants, interventions and outcomes

### Study setting {9}

Data will be collected from employees working in eligible UK organisations expressing an interest to MHFAE in undertaking MHFA training.

### Eligibility criteria {10}

Inclusion criteria are as follows: organisations expressing an interest (directly to MHFAE) in undertaking MHFA training, who have not undertaken this training previously and who agree to participate and are able to provide data on sickness absenteeism, presenteeism and other productivity data. For the purposes of this study, participants will be all employees working in each organisation who provide help-seeking outcome data.

Exclusion criteria are as follows: organisations who have already introduced MHFA across all sites and departments; those who decline to participate in adopting MHFA training; and organisations, employees, mental health first-aiders and recipients who participated in the pilot study.

The intervention will be delivered by qualified MHFAE trainers.

### Who will take informed consent? {26a}

The research team will elicit written agreement to participate from a relevant Senior Manager in each organisation. All employees within each included organisation will be invited to participate and be provided with a participant information sheet (PIS) providing detailed information and procedures regarding the study. The information provided will include the following: the benefits and potential harm of participation; how the team will collect, store and process data; and the participant’s right to withdraw from the study at any time, at least 2 weeks prior to any outcome assessment. Participant consent will be sought, using Qualtrics, before completing the online survey.

### Additional consent provisions for collection and use of participant data and biological specimens {26b}

N/A

### Interventions

#### Explanation for the choice of comparators {6b}

The control arm is termed as usual practice and is defined as an organisation that has not previously introduced MHFA and agrees to not introduce the 2-day MHFA training during the period of the study. The organisations allocated to usual practice arm will receive a brief consultation from MHFAE on the promotion of mental health and wellbeing in the workplace.

#### Intervention description {11a}

The MHFA intervention has three parts:

1. The 2-day MHFA training provided by MHFAE. This is a manualised training programme designed to provide individuals with the following: an in-depth understanding of mental health and the factors that can affect wellbeing, practical skills to spot the triggers and signs of mental health issues, confidence to step in, reassure and support a person in distress, enhanced interpersonal skills such as non-judgemental listening, knowledge to help someone recover their health by guiding them to further support—whether that is self-help resources, through their employer, the NHS, or a combination.
2. Raising awareness of the presence of MHFA in the organisation.
3. The application of MHFA to participants in the workplace. The MHFA training uses a manualised five-stage approach, ALGEE: Approach the person, assess and assist with any crisis; Listen and communicate non-judgementally; Give support and information; Encourage the person to get appropriate professional help and Encourage them to seek other forms of support.

MHFAE recommend training one employee in MHFA per ten employees; however, organisations are free to ask for any number of employees to be trained. The current study will record the ratio of participants trained in each organisation. No stipulation is made as to the selection of participants to be trained, and organisations are encouraged to advertise the training to all staff. The selection criteria used by each organisation will be assessed in the qualitative component of this study.

#### Criteria for discontinuing or modifying allocated interventions {11b}

A Trial Steering Committee (TSC), which will audit the conduct of the study, will have ultimate responsibility for deciding if the trial should be stopped on grounds of safety or efficacy. The occurrence of serious adverse events to participants will be assessed by the TSC and may be grounds for discontinuing the trial.

#### Strategies to improve adherence to interventions {11c}

A sample of MHFA training, staggered throughout the trial, will be observed by two independent researchers using structured observation forms to assess variability in fidelity between instructors, sites, and over time. This will ensure all interventions are being delivered as per protocol, to equally high standards, to improve participant adherence.

#### Relevant concomitant care permitted or prohibited during the trial {11d}

Organisations in the control arm must agree not to adopt MHFA training until after the study period.

#### Provisions for post-trial care {30}

Participants will be debriefed and provided with a written report of the outcomes containing contact details of the research group should they require further information.

### Outcomes {12}

#### Primary outcome

The primary outcome is Employees’ help-seeking behaviour at 6 months measured by the Actual Help-Seeking Questionnaire [AHSQ] [11]. The AHSQ measures help-seeking behaviour in the 2 weeks preceding the assessment and will be assessed at baseline, 6, 12 and 24 months. Help-seeking behaviour is determined by listing the number of help sources, the type of help sought and whether help seeking has occurred during the allocated time period.

The primary analysis will be conducted on those participants who receive MHFA and therefore is a per protocol analysis. Further analyses will examine the impact of the intervention at the organisational level using an intention-to-treat analysis with all respondents included.

As this is a small non-random subsample of randomised participants, it is recognised that this analysis will be vulnerable to selection bias and uncontrolled confounding. For this reason, a range of baseline participant characteristics will be included in the primary analysis to mitigate these limitations.

#### Secondary outcomes

All secondary outcomes and their measures are described in Table 1. In addition to these outcome measures, the researchers will also capture data on organisations’ sickness absence levels, presenteeism rates and measures of productivity.

**Table 1:** Data collection schedule and secondary outcome measures

Measure	Timing of measures				
	Baseline	Post-intervention (up to 3 months)	6 months	12 months	24 months
Demographics (age, gender, level of education, ethnicity, nature and frequency of and MHFA interventions directly experienced, etc.)	X				
Employees help-seeking intentions (General Help-Seeking Questionnaire) [12]	X	X	X	X	X
Employees’ mental health and wellbeing (Warwick-Edinburgh Mental health and Well-being Scale) [13]	X	X	X	X	X
Mental health first aiders’ mental health literacy (Mental Health Literacy Scale) [14]^a^	X	X	X	X	X
Mental health first aiders’ skills (ALGEE scoring system) [15]^a^	X	X	X	X	X
Employees’ quality of life (EQ-5D-5L and Short Form-12 Health Survey SF-12) [16]	X	X	X	X	X
Employees’ self-efficacy (Self-Efficacy for Seeking Mental Health Care Scale) [17, 18]	X	X	X	X	X
Employees’ social well-being (Social Well-being Scale) [19]	X	X	X	X	X
Employees’ use of standard mental health and other services (adapted version of the Client Services receipt Inventory) [20]	X	X	X	X	X

^a^Intervention group

### Assessment of cost-effectiveness

This component will answer the research question ‘Is MHFA more cost-effective than usual practice?’ This requires comprehensive estimates of the financial benefits derived from MHFA and its costs. Consequently, the evaluation will analyse the data from three perspectives reflecting the three main groups impacted by MHFA:

- Personal (employees’ quality of life)
- State/government (use of health and social services)
- Employers (staff productivity)

Data will be combined with unit cost data to provide a financial estimate of the change in service use observed. The results for those who had the MHFA training and those who did not will determine whether it is more cost-effective to purchase the training or not.

### Participant timeline {13}

#### Sample size {14}

**Fig. 1:**
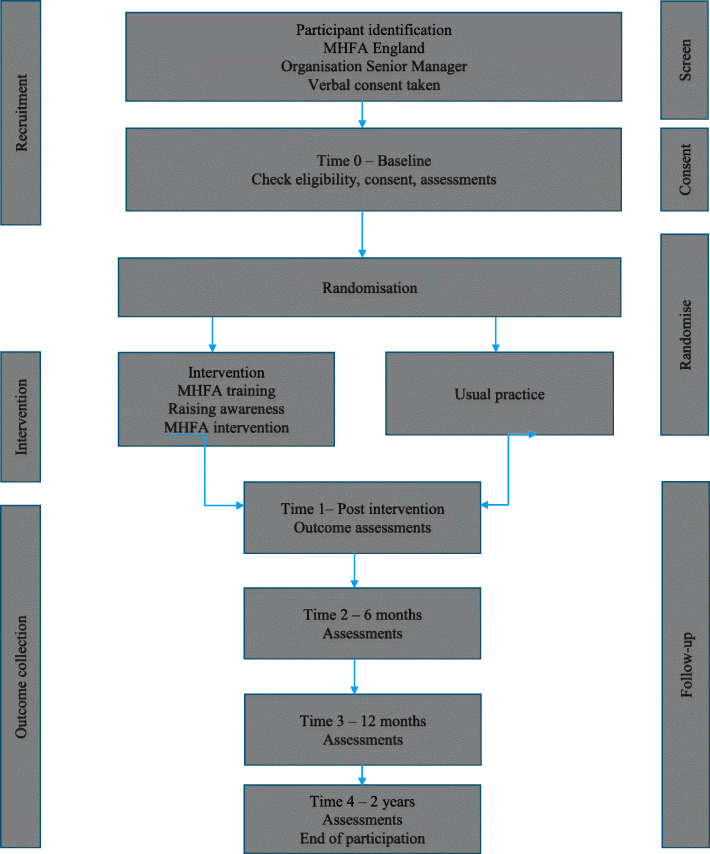
CONSORT trial flow diagram

The sample size calculation is based on a simplification of the proposed analysis model (Fig. 1).

To be able to detect a change of two additional help seeking resources [11], with 80% power at the 0.05 alpha level, assuming that the mean in the control group is 1 and in the intervention group 3 with a common standard deviation of 8, 506 participants are required, increasing to 596 allowing for 15% attrition at 6 months. Pilot data indicates a response rate of 40% across organisations and an average cluster size of 72 employees (calculated from organisations currently signed up) therefore approximately 29 employees per organisation (cluster size). With 24 clusters, 12 organisations per condition, and assuming an intraclass correlation coefficient (ICC) equal to 0.01 (ICC’s are lower for participant outcomes as opposed to process variables, when cluster size is large and when estimates are adjusted by participant baseline characteristics [21]), a minimum target sample size is 763, rounded up to 800, 400 per condition

#### Recruitment {15}

Organisations expressing an interest (directly to MHFAE) in undertaking MHFA training, who are willing to take part in the study, will be asked to contact Graham Durcan (GD: Centre for Mental Health) for detailed information about the study. Then, if willing to take part, the Senior Manager will sign a written agreement and their contact details will be sent to the research team. The research team will email the Senior Manager, detailing what will happen next, asking for company demographics (see secondary outcomes below) and requesting they send a standard email to ALL employees providing information about the study with a link to the survey for completion of baseline measures. One, 2 and 3 weeks later, the research team will send the Senior Manager another standard email, to be sent to ALL employees, as a gentle reminder to complete the survey.

At each follow-up, the research team will send participants (Senior Managers) up to four weekly email reminders to remind employees to complete measures.

**Assignment of interventions: allocation**

**Sequence generation {16a}**

**Concealment mechanism {16b}**

**Implementation {16c}**

#### Randomisation

The unit of randomisation is organisation, and randomisation of organisations will be stratified by size of organisation, with three strata being defined [22], small (< 50 employees), medium (50–249 employees) and large (> 250 employees). The randomisation schedule will be generated by GD using Random.org (https://www.random.org/randomness/) and allocation assigned to an organisation after completion of baseline measures.

### Assignment of interventions: blinding

#### Who will be blinded {17a}

Participants, mental health first aiders and researchers will not be blinded to treatment allocation due to the nature of the study. However, independent staff helping to process the data, the trial statistician and health economist will all be blinded to intervention status.

#### Procedure for unblinding if needed {17b}

N/A due to the nature of the trial.

### Data collection and management

#### Plans for assessment and collection of outcomes {18a}

Consent, baseline and follow-up data will be collected online, with the option for employees to complete them in hard copy should they be unable to complete them online. The research team will email the designated contact from each organisation, giving instructions and information for them to send to ALL employees and a link to the survey. One, 2 and 3 weeks later, follow-up emails will be sent to the same contact, asking them to send a reminder email, with a repeat survey link. After this period, participants who have not completed the survey will be treated as lost to follow-up.

#### Plans to promote participant retention and complete follow-up {18b}

Participants will be sent up to four weekly reminders to complete the survey at each time point.

### Data management {19}

Participant data will be exported from Qualtrics into SPSS version 22 by the research team (and any hard copies entered manually), stored on a password-protected network drive, will be accessible only to research team members and will only be linked directly with their participant ID code.

### Confidentiality {27}

Stored data will only be linked directly with the participant ID code. Any hard copies of data will be destroyed by confidential waste disposal 15 years after the research findings have been published. Electronic copies of data will be stored in two archives. In both cases, only anonymous data will be archived at London South Bank University archive and a national data repository.

### Plans for collection, laboratory evaluation and storage of biological specimens for genetic or molecular analysis in this trial/future use {33}

N/A as no biological specimens were collected as part of this trial.

### Statistical methods

#### Statistical methods for primary and secondary outcomes {20a}

All outcomes will be described with the appropriate descriptive statistics: mean and standard deviation for continuous outcomes (or medians and interquartile range for skewed data) and counts and percentages for dichotomous and categorical outcomes.

The analysis of the primary outcome will estimate the mean difference (with 95% confidence intervals) in the Actual Help-Seeking score at 6-month follow-up between the intervention (MHFA) and standard care groups using a mixed effects repeated measures model (which assumes incomplete outcome data to be missing at random). The model will incorporate demographic and other baseline covariates as fixed effects. The dependent variable will be the count of help sources on the Actual Help-Seeking questionnaire. The independent variable will be ‘time point’. The effect size of the intervention will be estimated as the exponentiated coefficient for the interaction term between time point (baseline versus 6-month follow-up) and intervention status (MHFA versus control group). A random effect of participant will be included in the model to adjust for the repeated measures on participants. ‘Employing organisation’ will be included as a higher level random effect (with participants nested in employers). Statistical significance will be at the 5% level.

Secondary outcomes will be analysed using an appropriate generalised linear model, for example binary logistic regression for dichotomous outcomes and ordinal logistic regression for ordered categorical outcomes. All models will be adjusted for employing organisation and baseline score (where applicable).

### Interim analyses {21b}

There will be no interim analyses.

### Methods for additional analyses (e.g. subgroup analyses) {20b}

Age, gender, level of education, ethnicity, nature and frequency of MHFA interventions directly experienced will be included in the primary analysis as covariates.

### Methods in analysis to handle protocol non-adherence and any statistical methods to handle missing data {20c}

The missing at random assumption for primary outcome data will be assessed further in sensitivity analyses. Treatment effects will be estimated under varying assumptions of data being missing not at random using pattern-mixture models. A complete case analysis will also be conducted.

### Plans to give access to the full protocol, participant-level data and statistical code {31c}

Access to the full protocol, participant-level data and statistical code will be available from the OSF.

### Oversight and monitoring

#### Composition of the coordinating centre and Trial Steering Committee {5d}

Graham Durcan from The Centre for Mental Health (CMH) has oversight of the project management on behalf of the funders. The Chief Investigator [CI] (Callaghan) has overall responsibility for the research.

A Research Management Group (RMG), which the CI will chair, comprising all authors, will advise and assist on the project’s management. The RMG meets every 6 weeks.

An Expert Reference Group (ERG), a group of independent research experts and lay people and the CI, will provide subject matter expertise to the funders and work with the CMH and London South Bank University’s representatives to guide and oversee the impact of the research. The ERG meets quarterly for the entirety of the project’s duration.

#### Composition of the data monitoring committee, its role and reporting structure {21a}

Data monitoring will be conducted by the Trial Steering Committee (TSC) comprising a trialist, statistician and health economist and will act as Data Safety Monitoring Board (DSMB). The TSC will oversee the conduct of the trial independently of and on behalf of the project funders and sponsors and ensure it is carried out with reference to good practice. The TSC will have ultimate responsibility for deciding if the trial should be stopped on grounds of efficacy. The TSC is a sub-group of the project Expert Reference Group (ERG) set up by the funders and will audit the trial conduct through reports submitted to it by the researchers on a three-monthly basis.

#### Adverse event reporting and harms {22}

We anticipate little risk to participants in the study. The Senior Manager in each company will be asked to report to the CI in writing any adverse or untoward incidents that occur because of the study. The CI will assess each adverse event and decide on an appropriate course of action. In case of serious adverse events (SAEs), i.e. those causing serious injury or death, the CI will notify the Ethics Committee and the TSC as soon as possible after being notified. A TSC within the ERG consisting of members independent of the investigators, their employing organisations, funders and sponsor will monitor trial progress and conduct and will have ultimate responsibility for deciding if the trial should be stopped on the grounds of safety.

#### Frequency and plans for auditing trial conduct {23}

The Trial Steering Committee (TSC) will audit the trial conduct.

#### Plans for communicating important protocol amendments to relevant parties (e.g. trial participants, ethical committees) {25}

Important changes to the protocol will be reported to the funders, the trial registry, the TSC and the ethics committee. The latter will need to approve any changes.

#### Dissemination plans {31a}

We will engage potential adopters throughout the life of the project to enable transfer of project outcomes and effective pathways to impact through educational outreach visits to companies and other services and interactive educational meetings through discussion forums involving the research team and local opinion leaders in each site to influence dissemination. We will publish papers in high impact journals, professional magazines, newsletters of Non-Governmental agencies and presentations to policy makers, roundtable discussions and a final conference. We will create and maintain a project website and establish a newsletter, monthly blogs and a quarterly update bulletin diffused via various social media.

## Discussion

The study is the first to evaluate the effect of MHFA in the workplace on employees with direct and indirect experience of the intervention, when compared with usual practice. Being also the first to assess, systematically, the social impact of MHFA and investigate its cost-effectiveness adds to the originality of the study. The study promises to yield important data, as yet unknown, regarding the effectiveness, cost-effectiveness, implementation issues and the sustainability of MHFA in the workplace.

## Trial status

Participants are being actively recruited for this trial. Recruitment for the trial began in January 2020 and is expected to continue until January 2021. This manuscript describes Version 18 of the study protocol, dated 24 June 2020.

## Abbreviations

EMPOWER: Evaluation of Mental Health First Aid from the Perspective Of Workplace End UseRs
MHFA: Mental Health First Aid
MHFAE: Mental Health First Aid England
RCT: Randomised controlled trial
ALGEE: Approach the person, assess and assist with any crisis; Listen and communicate non-judgementally; Give support and information; Encourage the person to get appropriate professional help and Encourage them to seek other forms of support
AHSQ: Actual Help-Seeking Questionnaire
EQ-5D-5L: Employees’ Quality of Life
SF-12: Short Form-12 Health Survey
ID: Identification
TSC: Trial Steering Committee
CI: Chief Investigator
RMG: Research Management Group
ERG: Expert Reference Group
DMC: Data monitoring committee
SAEs: Serious adverse events
